# Riemannian Spatio-Temporal Features of Locomotion for Individual Recognition

**DOI:** 10.3390/s19010056

**Published:** 2018-12-23

**Authors:** Jianhai Zhang, Zhiyong Feng, Yong Su, Meng Xing, Wanli Xue

**Affiliations:** College of Intelligence and Computing, Tianjin University, Tianjin 300350, China; zyfeng@tju.edu.cn (Z.F.); suyong@tju.edu.cn (Y.S.); xingmeng@tju.edu.cn (M.X.); xuewanli@tju.edu.cn (W.X.)

**Keywords:** individual recognition, Riemannian manifold, spatio-temporal representation, Riemannian mean motion

## Abstract

Individual recognition based on skeletal sequence is a challenging computer vision task with multiple important applications, such as public security, human–computer interaction, and surveillance. However, much of the existing work usually fails to provide any explicit quantitative differences between different individuals. In this paper, we propose a novel 3D spatio-temporal geometric feature representation of locomotion on Riemannian manifold, which explicitly reveals the intrinsic differences between individuals. To this end, we construct mean sequence by aligning related motion sequences on the Riemannian manifold. The differences in respect to this mean sequence are modeled as spatial state descriptors. Subsequently, a temporal hierarchy of covariance are imposed on the state descriptors, making it a higher-order statistical spatio-temporal feature representation, showing unique biometric characteristics for individuals. Finally, we introduce a kernel metric learning method to improve the classification accuracy. We evaluated our method on two public databases: the CMU Mocap database and the UPCV Gait database. Furthermore, we also constructed a new database for evaluating running and analyzing two major influence factors of walking. As a result, the proposed approach achieves promising results in all experiments.

## 1. Introduction

Individual identification from locomotion or action is a central problem of computer vision, which has attracted ever-increasing attention for its applications in biometrics and surveillance. This is due to the fact that (a) human locomotions recorded by cameras are non-contact, non-invasive, and non-cooperating, in contrast with the other biometric identification technologies, such as face, fingerprint, DNA, or iris recognition [1]. Moreover, (b) several studies have proved that more than 20 different biological characteristics in human motion are unique for each individual [2]. In the well-known psychology test of point light displays, the human in motion could be rapidly perceived from points of light attached to body joints [3]. The later research has shown that the different types of locomotion could also be discriminated [4,5]. Particularly, humans can recognize their friends or unfamiliar people under brief exposures from the motion of joints [6,7]. Therefore, locomotion-based individual identification has become flourishing in the computer vision community recently.

The observational locomotions are commonly consecutive RGB-based images or depth images. The main challenge arises from the variations in illumination, views, and appearance. Thus, the various approaches have been proposed to alleviate these influences. For example, a cross-view approach has been proposed to reconstruct the 3D structure from a 2D video sequence [8]. However, these 2D image-based approaches need to explore complicated models against many variations. The results, sometimes, are not satisfying. In contrast, with the development of optical motion capture system and pose estimation techniques, a 3D skeletal sequence is more reliable for two reasons. Firstly, it eliminates many variations mentioned above in a sense that the variations caused by irrelevant factors, such as illumination, appearance and background etc., are never considered any longer. Secondly, each individual has a unique bio-mechanical motion pattern, which heavily depends on body weight, muscular strength, and tissue flexibility. Therefore, we can infer that the 3D joint trajectories have enough discriminative information for individual identification.

In this article, we design a study focused solely on walking and running, which is an integral part of many day-to-day behaviors. They have many advantages, such as periodicity and uniqueness, which make it suitable for recognizing different individuals. It is worth noting that there is an essential difference between individual recognition and human action recognition. In the field of individual identification, the focus is on identifying personal styles of a certain movement. In contrast, the aim of human action recognition is opposite, generalizing the actions with the variations of different people.

**Figure 1:**
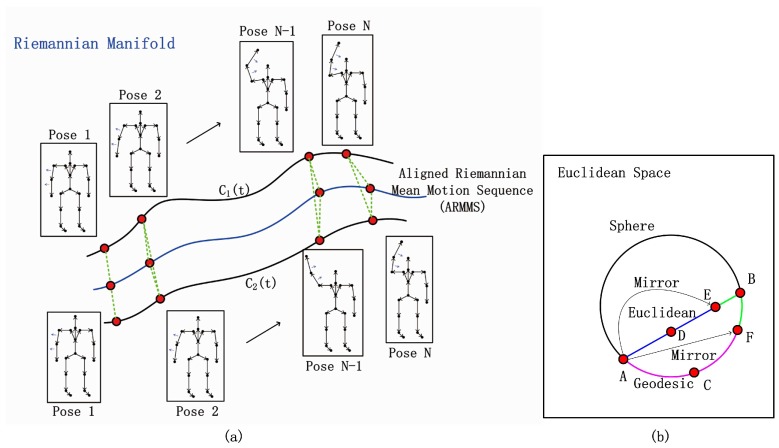
Illustration of the proposed (**a**) aligned Riemannian mean motion sequence (ARMMS). (**b**) A and B are the pair of poses in two different sequences $C_{1}\left(t\right)$ and $C_{2}\left(t\right)$. C and D are the mean points on the sphere and plane, respectively. F and E are mirrors of A for manifold and Euclidean cases, respectively. The difference between A and B in the green on the sphere is larger than in the case on the Euclidean plane, i.e., geodesic $\overset{⌢}{BF}>BE$.

Three observations motivate us to consider Riemannian features: (a) The skeletal structures of different people are unified on the Riemannian manifold in order to fairly use collected data. (b) The human skeleton represented on a Riemannian manifold has a specific topological structure that is able to reflect real and rich information compared to Euclidean space. (c) A certain movement from different individuals can be approximately modeled by a unified dynamic system on Riemannian manifold [9], and the real difference between the individuals can be measured by the intrinsic geodesic distance against the mean system. Moreover, the features of the Riemannian manifold are more discriminative than the features on Euclidean space. This is due to the fact that the difference derived by geodesic along the manifold is larger than the difference in the Euclidean metric, which is illustrated in Figure 1b. In particular, the enhancement of subtle differences will essentially facilitate the tasks of individual recognition.

**Figure 2:**
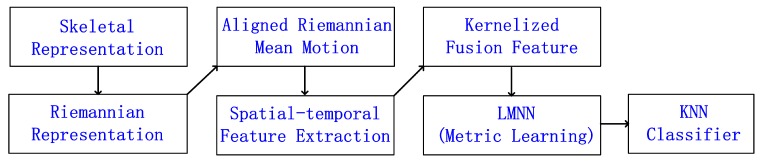
Overview of the proposed framework.

In this paper, we develop Riemannian geometric features on locomotions for individual identification. The proposed approach models the state space of joints on the Riemannian manifold and obtains an aligned Riemannian mean motion sequence (ARMMS), as shown in Figure 1a. The unique spatial feature of each individual is naturally described by the geometrical distance between sequences and ARMMS. We then encode the unique temporal variations of spatial features by the temporal hierarchy of covariance descriptors. In order to model the nonlinear feature relationship in an efficient manner, the spatial and the temporal features are mapped in high-dimensional feature spaces by the radial basis function (RBF) kernel. Finally, we utilize a kernel metric learning strategy to improve the classification accuracy. The proposed 3D spatio-temporal geometric feature representation based on Riemannian manifold obtains excellent results for individual recognition on both walking and running sequences. The framework is shown in Figure 2.

The contribution of the proposed work is threefold: Firstly, we propose efficient spatial and temporal features on the Riemannian manifold, which have rich statistical information and enhances the subtle differences between different individuals. Secondly, we demonstrate the effectiveness and robustness of the proposed approach by conducting extensive experiments on the sequences captured from three different sensors (the Vicon system, Kinect, and a digital camera). Finally, we also built a locomotion database for exploratory factor analysis and recognizing individuals based on walking or running.

The rest of this paper is organized as follows. Section 2 presents a review of related work. The proposed framework is described in Section 3, followed by an experimental evaluation of our method in Section 4. Finally, Section 5 concludes the paper.

## 2. Related Work

Individual recognition has received ever-increasing attention as a remote biometric identification technology. Most existing techniques have been developed to recognize individuals by their walking or running fashions (gait). The current methods for gait recognition can be classified into two categories: appearance-based approaches and model-based approaches.

One of the earliest appearance-based approach can be seen in [2], they build a descriptor by a binarized silhouette from a side view to recognize each individual. The binary silhouette feature has been also used to train a probabilistic model [10]. In addition, the authors of [11] present a gait recognition method to compute a gait energy image (GEI) descriptor, which is the average of all silhouette images for a single gait cycle. Inspired by the GEI, the authors of [12] introduced the depth energy image (DEI), which is the average of the depth silhouettes taken along a gait cycle. Similar work also include chrono-gait images (CGIs) [13], gait flow images (GFIs) [14], and gait energy volume (GEV) [15]. Although [16] has shown that the GEI required less computational effort and could achieve a stable result for gait recognition, the appearance is easily camouflaged. Moreover, appearance changes caused by the variations in viewing angle would cause difficulties for most of the appearance-based approaches. This problem cannot be easily avoided in practical applications [17,18,19,20].

The second category is model-based approaches, which have better performance in the case of view variants [8,21]. Several approaches are proposed to construct 3D gait information through multiple calibrated cameras [22,23]. The authors of [22] apply an image-based rendering on a 3D VH model to reconstruct gait features. The authors of [23] propose an approach to estimate the observation angle at each frame from the walking direction, and images are synthesized from 3D reconstructions. In addition, several approaches extract gait features that are robust to viewing changes [24,25]. The authors of [24] propose a method based on homography to compute view-normalized trajectories of body parts. A self-calibrating view-independent recognition approach is proposed in [25], where lower limbs are estimated based on markerless motion estimation and then reconstructed in the sagittal plane. The model-based approaches can achieve promising performance. However, the dependence on the multiple calibrated cameras under fully controlled and co-operative environments makes these approaches hard to be implemented in a real-world environment. Moreover, the performance of gait recognition decreases as the viewing angle increases.

In recent years, human representations based on 3D skeleton data have been attracting an increasing amount of attention. Compared with 2D visual data, additional depth information provides more geometric information of motion data. The recent development of deep learning approaches make it easier to obtain 3D human poses from a single view or video sequence [26,27,28]. These approaches train their models by sequences captured from various motion capture (Mocap) databases (such as Human 3.6 M [29] and KTH [30]). The human skeleton representation can disentangle identity-unrelated factors, such as illumination conditions, dressing, and camera viewpoints, which make it more suitable for representing the discriminative information of individuals. The authors of [31] compute distance–time dependency signals on the the motion sequences selected from the CMU Mocap database, which express the distance variation between two specific joints over time. The authors of [32] extracted a couple of joint angles from two signature poses within a gait cycle to form a gait pattern descriptor, and classified the query subject by the baseline 1-Nearest Neighbor. It can well handle both the variations in joints and the pairwise relationship between joints. These approaches make better use of biometric cues than do the appearance-based approaches. However, these features are not discriminative enough to explain the essential difference between individuals. It should be noted that, as static features, each skeleton is unique. Using this trivial static, skeletal feature itself can still result in perfect recognition. Therefore, untreated position data of joints potentially including the precise static features would make the experiment meaningless. In order to use the Mocap data accurately and fairly, in [32], a prototypical skeleton was constructed and used to represent the bodies of all subjects, and [33] used the angle rotation features to disable static features. In our approach, we model the human poses on the Riemannian manifold, which is a unified representation of the skeleton. This representation has been used in human pose tracking and estimation approaches [9,34].

## 3. Our Method

In this section, we will introduce the formulations of Riemannian spatio-temporal features. Furthermore, the kernel metric learning method which is used for feature classification will also be depicted in detail.

### 3.1. Preliminary

Now we present three steps for pre-processing motion sequences: period extraction, alignment, and interpolation. Since human locomotion is a kind of periodic signal, a movement sequence may include several cycles. Capturing a periodic cycle means starting and stopping the sample extraction cycle when certain movements are detected. We extract action cycles by utilizing the speed auto-correlation function of ankle or toe (depending on the structure of the skeleton). This is because the foot is the first contact part with the ground and it moves up the kinematic chain. The speed auto-correlation function possessing the same cycle as the original signal can be utilized to eliminate the influence of the stochastic fluctuations from the signals [35]. The speed of a certain joint is the changing rate of its position in Euclidean space $v=\left|\vec{v}\right|=\left|\frac{ds}{dt}\right|$. A speed auto-correlation coefficient *R* is defined as

(1) $$R=\frac{1}{F-1-\left|\Delta f\right|}\sum_{i=1}^{F-1-\left|\Delta f\right|}v_{i}v_{i+\Delta f}$$

where the lag parameter Δf is the phase shift in the number of samples, and *F* is the number of frames. The speed auto-correlation function is represented by a sequence of auto-correlation coefficients over increasing time lags. Since phase shifts can be performed with identical results in both positive and negative directions, the speed auto-correlation curve can be plotted symmetrically with the zeroth shift located centrally. For a time series of speed signals during walking or running, each cycle can be represented as phase shifts between peak values of auto-correlation coefficients [36].

Similar movements performed by the same or different subjects can have different execution rates in a periodic process. To compensate for these unsynchronized sequences, we use generalized time warping (GTW) proposed by [37], which could find an optimal alignment between many multi-dimensional time series of different modalities. However, the GTW reproduces multiple identical frames for alignment purpose, which is obviously contrary to the law of movement. In order to ensure continuity and smoothness of the motion trajectory, the same frames are replaced by interpolating on Riemannian manifold.

An articulated body can be described by a tree-structure open kinematic chain [38], which refers to an assembly of rigid bodies connected by joints to provide constrained motion. In order to obtain richer statistical information about the spatial structure of the poses, we use the orientation of the link between joints i,j. Subsequently, they are represented as a series of vectors on a sphere $x_{f}^{ij}=\frac{x_{f}^{i}-x_{f}^{j}}{{∥x_{f}^{i}-x_{f}^{j}∥}_{2}}\in S^{2}$. Based on the kinematic chain, the whole pose $x_{f}\in M$ can be represented as the Cartesian product of all the relative rotations between consecutive joints [34]:

(2) $$M=S^{2}\times S^{2}\times \ldots \times S^{2}.$$

For brevity, in the following, we denote the joint state $x_{f}^{ij}$ on the Riemannian manifold as $x_{f}^{i}$. The same frames $\left(x_{f+1},\ldots x_{w-1}\right)$ between two different frames $x_{f}$ and $x_{w}$, are revised by uniformly interpolating for each joint *j* (j=1,…,J) on a Riemannian manifold, where *J* is the number of joints. Specifically, for joint *j*, the uniform interpolation takes place on the geodesic between $x_{f}^{j}$ and $x_{w}^{j}$. First, we use the logarithm operation in Equation (3) to linearize the geodesic between $x_{f}^{j}$ and $x_{w}^{j}$ on the tangent space of $x_{f}$:

(3) $$v_{f}^{j}=\log_{x_{f}^{j}}\left(x_{w}^{j}\right)$$

where $v_{f}^{j}$ is the tangent vector of $x_{f}^{j}$. The tangent vector is then cut into w−f equal parts $\left(v_{f+1}^{j},\ldots ,v_{w-1}^{j}\right)$. Finally, we use the exponential mapping in Equation (4) to obtain the revised results $\left({\overset{⌢}{\;x}}_{f+1}^{j},\ldots ,{\overset{⌢}{\;x}}_{w-1}^{j}\right)$ on the manifold.

(4) $${\overset{⌢}{\;x}}_{f+1}^{j}=\exp_{x_{f}^{j}}(v_{f}^{j}).$$

### 3.2. Riemannian Geometric Features Representation

Individual recognition aims to identify the unique motion pattern of each individual by biometric cues. The active appearance model (AAM) states that the most important aspect of encoding for facial identity is the information about an individual face *x* relative to the mean face *m* [39]:

(5) x=m+Cb

where *C* is a set of orthogonal modes of shape variation, and *b* is a set of shape parameters. However, for the articulated objects with properties of high dimensional and nonlinearity, the formulation in Equation (5) cannot model the variations in human pose.

Similar to AAM, we tend to obtain a **mean motion sequence** on the Riemannian manifold, which can be utilized to extract the discriminating features for describing individual motion pattern. To achieve this objective, for each frame of all sequences, we define a **mean pose**, which is composed of the geometric means of all joints. The mean value of a set of points on a manifold lying on a small neighborhood of manifolds can be calculated intrinsically. We use the Karcher mean [40], which is defined as the point on the manifold that minimizes the sum of squared distances. The Karcher mean of joint *j* in frame *f* can be represented as

(6) $$m_{f}^{j}=\arg\min_{m_{f}^{j}}\sum_{s=1}^{S}d^{2}\left(m_{f}^{j},x_{f}^{j}\left(s\right)\right)$$

where *S* is the number of sequence.

Finally, the mean pose composed by *J* joints in frame *f* can be denoted as ${\bar{x}}_{f}={\left\{m_{f}^{j}\right\}}_{j=1}^{J}\in R^{J}$, and the mean motion sequence containing *F* mean poses is defined as ${\left\{{\bar{x}}_{f}\right\}}_{f=1}^{F}\in R^{JF}$. Algorithm 1 describes the steps to compute mean sequence. Given a skeleton sequence ${\left\{x_{f}\right\}}_{f=1}^{F}\in R^{JF}$ of a certain individual, the most direct way to characterize the differences between individuals is to calculate the geometric distance against the mean sequence. For a vector $x_{f}^{j}$ of joint *j*, the geodesic distance $G_{f,j}$ can be obtained by

(7) $$G_{f,j}\left(x_{f}^{j},m_{f}^{j}\right)={\left[\left(\log_{m_{f}^{j}}x_{f}^{j}\right){\left(\log_{m_{f}^{j}}x_{f}^{j}\right)}^{T}\right]}^{\frac{1}{2}}.$$

Finally, the Riemannian geometric features in frame *f* can be represented as a vector $G_{f}\in R^{J}$. As a result, the geometric features of sequence *s* (s=1,…,S) containing *F* frames can be denoted as a feature vector $G\left(s\right)\in R^{FJ}$.

**Algorithm 1** Computing mean sequence.**Input:** a set of sequences $\left\{x_{f}\left(s\right)\right\}$, s=(1,…,S) and $\varepsilon_{1},\varepsilon_{2}>0$**Initialization:**$m_{f}^{j}\leftarrow x_{f}^{j}$, j=(1,…,J) and f=(1,…,F) **for**
f=1 to *F*
**do**  **for**
j=1 to *J*
**do**   **while**
$\left|{\bar{v}}_{f}^{j}\right|<\varepsilon_{1}$
**do**    Compute $v_{f}^{j}\leftarrow \log_{m_{f}^{j}\left(i\right)}\left(x_{f}^{j}\left(s\right)\right)$, for s=1,…,S    Compute mean tangent vector ${\bar{v}}_{f}^{j}\leftarrow \frac{1}{S}\sum v(s)$    Update $m_{f}^{j}\left(i\right)$: $m_{f}^{j}\left(i+1\right)\leftarrow \exp_{m_{f}^{j}\left(i\right)}\left(\varepsilon_{2}{\bar{v}}_{f}^{j}\right)$    i=i+1   **end while**   **Return:** mean joint $m_{f}^{j}$  **end for**  **Return:** mean pose ${\bar{x}}_{f}={\left\{m_{f}^{j}\right\}}_{j=1}^{J}$ **end for** **Return:** mean sequence ${\left\{{\bar{x}}_{f}\right\}}_{f=1}^{F}$

### 3.3. Riemannian Temporal Hierarchy of Covariance Descriptors

**Figure 3:**
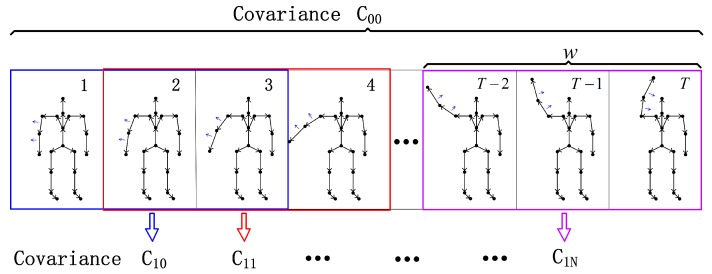
Temporal construction of the covariance descriptor.

The covariance descriptor has also been used for the tasks of human motion analysis. For human action recognition, the pose variations within a sequence is encoded as a sample covariance descriptor [41,42]. Since the symmetric positive definite (SPD) matrices can be treated as points on the Riemannian manifold, they deal with classification tasks by nearest neighbor (NN) using the log-Euclidean metric. In addition, manifolds can be also mapped to a reproducing kernel Hilbert space (RKHS) via the kernel trick [43,44,45]. However, the elements in the covariance descriptor contain the unique skeleton information of each individual, and this descriptor does not enclose temporal evolution information of motion sequences. Therefore, the traditional method cannot be used directly in the task of individual identification. In order to encode the temporal dependency of geometric features over time, we use hierarchical covariance matrices, each covering a sub-sequence of the input sequence. Different from Cov3DJ proposed by [46], which models variations in the joints in Euclid space, the proposed approach encodes the dynamic variations of Riemannian geometric features into hierarchical covariance matrices. For each sequence, the covariance matrix can be described as a symmetric matrix $C\left(\cdot ,\cdot \right)\in R^{J\times J}$. The covariance matrix of the range of frames from $f_{1}$ to $f_{2}$ can be calculated as follows:

(8) $$C\left(f_{1},f_{2}\right)=\frac{1}{f_{2}-f_{1}-1}\sum_{f=f_{1}}^{f_{2}}\left(G_{f}-{\bar{G}}_{f}\right){\left(G_{f}-{\bar{G}}_{f}\right)}^{T}$$

where ${\bar{G}}_{f}$ is the sample mean of $G_{f}$, and the *T* is the transpose operator. The upper triangle of the covariance matrix in this case is J(J+1)/2, which is the length of each sub-descriptor. As a typical experimental setup also used in [46], we use a two-level descriptor and allow overlap in the second levels to construct the temporal covariance descriptors. The hierarchical construction is shown in Figure 3. The top level $C_{00}$ is computed over the entire motion sequence. The lower levels $\left[C_{10}\ldots C_{16}\right]$ are computed over smaller windows, overlapping the entire sequence.

Figure 3 shows a covariance descriptor with *J* joints, resulting in a descriptor for each sequence $C\left(s\right)\in R^{\frac{7J(J+1)}{2}}$ (s=1,…,S). For a more detailed and rigorous treatment, we refer the reader to the standard texts [46].

### 3.4. Kernel Metric Learning

As a result from the previous two stages, for each training sample *n*(n=1…N), we can obtain the spatial geometric feature $G(n)\in R^{FJ}$ and the temporal hierarchy feature $C(n)\in R^{\frac{7J(J+1)}{2}}$. The objective of the feature fusion stage is to combine spatial and temporal information that improves individual recognition accuracy. The spatio-temporal features for all sequences contains the unique motion cue of each individual. However, the variation in human motion space is highly nonlinear. The performance of the large margin nearest neighbor (LMNN) [47] might degrade when the given data has a nonlinear structure. Therefore, we utilize a kernel metric learning approach to learn a low-rank distance metric in a high dimensional feature space. The metric learning approach is inspired by the kernel extension of the LMNN (KLMNN) algorithm proposed by [48,49], which is known to deliver a significant performance improvement in the recognition system. Firstly, we introduce the kernel trick to map the input spatial and temporal features to high-dimensional feature spaces. Usually, the kernel function can be written as

(9) $$k\left(x_{i},x_{j}\right)=\Phi {\left(x_{i}\right)}^{T}\Phi \left(x_{j}\right)$$

where Φ(·) is an implicit nonlinear mapping, and k(·,·) is the induced kernel function.

In this context, for the spatial geometric and the temporal hierarchy features, we use the radial basis function (RBF) kernel:

(10) $$k_{s}\left(G\left(i\right),G\left(j\right)\right)=\exp\left(-\frac{{∥G(i)-G(j)∥}^{2}}{2\delta^{2}}\right)$$

(11) $$k_{t}\left(C\left(i\right),C\left(j\right)\right)=\exp\left(-\frac{{∥C(i)-C(j)∥}^{2}}{2\beta^{2}}\right)$$

where δ and β are estimated as the mean of the training data pairwise distances.

After obtaining the kernelized spatial geometric and temporal hierarchy features, here we introduce a fusion scheme based on the Hadamard product of kernel functions. In particular, the pairwise similarity matrix for the all sequences is defined as the element-wise multiplication between two kernels $k_{st}=k_{s}⊗k_{t}\in R^{N\times N}$. The *n*-th column of the kernel matrix $k_{st}$ is a feature vector of the *n*-th sequence, which, for brevity, is denoted by $k_{st}\left(n\right)$. Instead of computing simple Euclidean distance, we use the Mahalanobis distance metric to measure the distance between these kernelized features. Considering the k−NN classifier, the loss function is a measurement of violations made by impostor samples and distancing among target neighbors. The distance between two target neighbors $k_{st}\left(i\right),k_{st}\left(j\right)\in R^{N}$ that we want to be closest to each other, is defined as

(12) $$D\left(k_{st}\left(i\right),k_{st}\left(j\right)\right)={∥L\left(k_{st}\left(i\right)-k_{st}\left(j\right)\right)∥}_{2}^{2}.$$

On the other hand, we want the distance between the $k_{st}\left(i\right)$ and the impostor $k_{st}\left(l\right)$, which is closer to $k_{st}\left(i\right)$ without being targets, to keep away from each other in the function of linear transformation *L*. Therefore, in our model, the loss function is represented by both push and pull components, trying to pull the targets and push the impostors simultaneously. Meanwhile, a penalty term ${∥L^{T}L-I∥}_{F}$ is imposed to maintain orthogonal independence amongst the different coordinates of the metric space. It is defined as

(13) $$\begin{array}{ccc} \varepsilon (L) & = & \mu \sum_{i,j\to i}\sum_{i,l↛i}\left[D(k_{st}\left(i\right),k_{st}\left(j\right))-D(k_{st}\left(l\right),k_{st}\left(i\right))+\zeta \right]\\ & + & \left(1-\mu \right)\sum_{i,j\to i}\left[D(k_{st}\left(i\right),k_{st}\left(j\right))\right]+\lambda {∥L^{T}L-I∥}_{F} \end{array}$$

where the notations j→i and l↛i denote that *j* is the index of targets of sample *i*, while *l* is the index of impostors of sample *i*. The key idea is to learn the transformation *L* to be a non-square matrix of size p×N, with p≤N. Thus, *L* defines a mapping from the high-dimensional space to a low-dimensional embedding. In addition, the final regularization term in Equation (13) enforces that the equivalent metric $L^{T}L$ should remain close to the identity matrix. The λ and μ are the weighting parameters.

The optimal transformation *L* that minimizes Equation (13) can be found by solving the global optimization problem:

(14) $$L^{*}=\arg\min_{L}\varepsilon \left(L\right).$$

In order to solve Equation (14) by the gradient descent approach, we compute the derivative term of ε(L), as follows:

(15) $$\begin{array}{ccc} \frac{1}{2}\frac{\partial \varepsilon }{\partial L} & = & L\sum_{i,j\to i}\sum_{l↛i}\left[M_{i,j}-M_{i,l}\right]\\ & + & 2\lambda L\left(L^{T}L-I\right) \end{array}$$

where $M_{i,j}=\left(k_{st}\left(i\right)-k_{st}\left(j\right)\right){\left(k_{st}\left(i\right)-k_{st}\left(j\right)\right)}^{T}$ and $M_{i,l}=\left(k_{st}\left(i\right)-k_{st}\left(l\right)\right){\left(k_{st}\left(i\right)-k_{st}\left(l\right)\right)}^{T}$.

For giving a reasonable initialization before starting the iterations, in the feature learning stage, we use linear discriminant analysis (LDA) [50] to initialize *L* on the feature matrix. The optimization is performed until the maximal number of iterations is reached or the gradient below a set threshold value. In our experiment, we learn the transformation $L\in R^{p\times N}$ with maximal epoch=40 and the threshold value δ=0.01.

## 4. Experiments

In this section, we present the results of individual identification tasks on three databases. Here, two cases are considered: (a) the 3D locations of the joints are provided (Section 4.2), and (b) the 3D joint locations are unknown (Section 4.3). In every experiment, the procedure is repeated 10 times and the accuracy is averaged. The parameters are set as λ=0.1 and μ=0.2. On all databases, i.e., CMU, UPCVgait, and our database, the classification performances are obtained by means of stratified five-fold cross-validation. Four pieces are used for training, and one piece is used for testing. For the compared methods in CMU and UPCV, we stimulate the feature extraction and use a unified classifier for experiments.

### 4.1. Database

In our experiments, two publicly available databases are exploited to evaluate our approach: the CMU Mocap database [51] and the UPCV Gait database [52].

For the CMU database, the experimenter wore a black jump suit with 41 markers, and motions were recorded with an optical marker-based Vicon system. The video surveillance environment of 30 m${}^{2}$ was surrounded by 12 cameras with a sampling rate of 120 Hz at heights ranging from 2 to 4 m. Highly accurate 3D poses were obtained in the form of relative body point coordinates in each video frame of the motion sequences. Each pose was stored in a bone-rotational form, which is a generic representation method for describing the deformation of marked joints over time.

The UPCVgait database consists of data with respect to walking sequences from 30 individuals (15 males and 15 females), which targets pose-based gender and identity recognition tasks. For each individual, there were five sequences captured during a small time cycle. Each person was asked to walk in a straight line through a corridor at a normal speed, and the Kinect sensor was placed 1.70 m above the ground, at the left of the walking path, with the sensor’s principal direction at an angle of $30^{\circ }$ relative to the walking line. The length of sequence ranges from 55 to 120 frames, depending on the walking speed of each individual. Poses with 20 key joints were estimated using Microsoft Kinect SDK at approximately 30 fps.

### 4.2. Evaluation with Known 3D Poses

**Figure 4:**
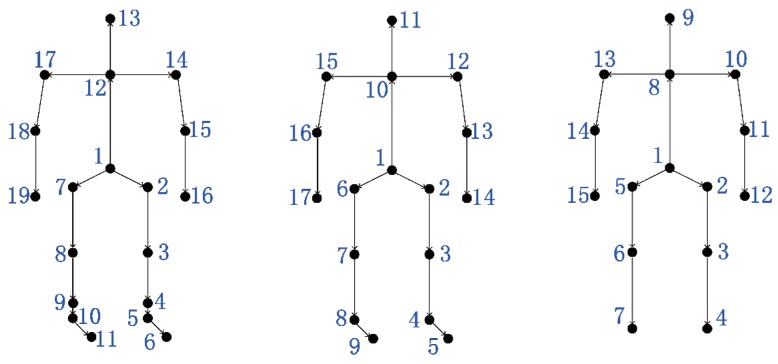
Skeletal structures: **left**: the CMU Mocap database, **middle**: the UPCVgait database, and **right**: our database.

The CMU Mocap database contains an extensive collection of data with respect to walking sequences of different subjects. However, the length of sequences varies largely, which may lead to sample imbalance, and there was no unified rule for walking process. In this experiment, subjects with more than 20 cycles and who walked straight were chosen. We select seven walking subjects (#7, #8, #12, #15, #16, #35, #39) that performed 140 walking cycles in total, with 20 cycles per subject. As shown in Figure 4(**left**), we used a skeleton with 19 joints. To quantitatively evaluate the performance, we compared our method with seven baselines [31,53,54,55,56,57,58].

As shown in Table 1, the proposed approach achieves a 98.70% classification rate, exceeding the second-best approach by more than 2%. Our results in this table correspond to a feature dimension of p=112 and 150 frames per cycle in the alignment stage. This result proves that the proposed feature representation can reveal the unique spatio-temporal characteristics of each individual. Moreover, the proposed approach models individual motion pattern by measuring the distance from the mean motion sequence, which is more distinguishable and reliable than other compared approaches.

**Figure 5:**
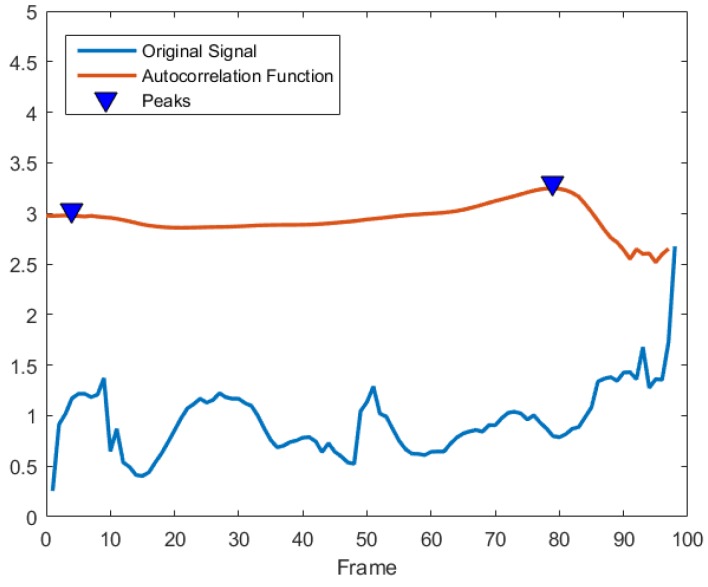
Motion cycle extraction by autocorrelation function.

In the UPCVgait database, we used the skeleton with 17 joints as shown in Figure 4(**middle**). The distorted poses in each sequence were removed in the cycle extraction stage. This is because, as shown in Figure 5, the distorted poses at the end of a sequence would show weak correlation to other poses. Since the frame rate of this database is lower than the CMU Mocap database, we set 50 frames per cycle in the GTW alignment stage. To quantitatively evaluate the performance, we compared our method with five baselines [53,57,59,60,61]. Among these methods, the authors in [59] applied the covariance and its dissimilarity measure concept on the skeletal trajectories. Similarly, the authors in [61] fused information from feature representations from both Euclidean and Riemannian spaces. One of the feature they used was the kernelized covariance matrix. Compared with the vector of locally aggregated descriptors (VLAD) encoding method used in [61], the proposed features not only preserve the temporal information but also depict intrinsic distance on the Riemannian manifold. As shown in Table 2, our approach achieves a 97.90% classification rate, exceeding the second-best approach [61] by more than 1%. Our results in this table correspond to a feature dimension of p=110.

### 4.3. Evaluation with Unknown Poses

In practical applications, such as video surveillance and forensics investigation, it is very difficult to obtain real 3D human motion data. Moreover, the current available walking Mocap databases are rather small and consider little about the factors that contribute to motion performance. Contrary to the above databases captured by special sensors, we built a database that records the walking and running sequences by digital camera.

**Figure 6:**
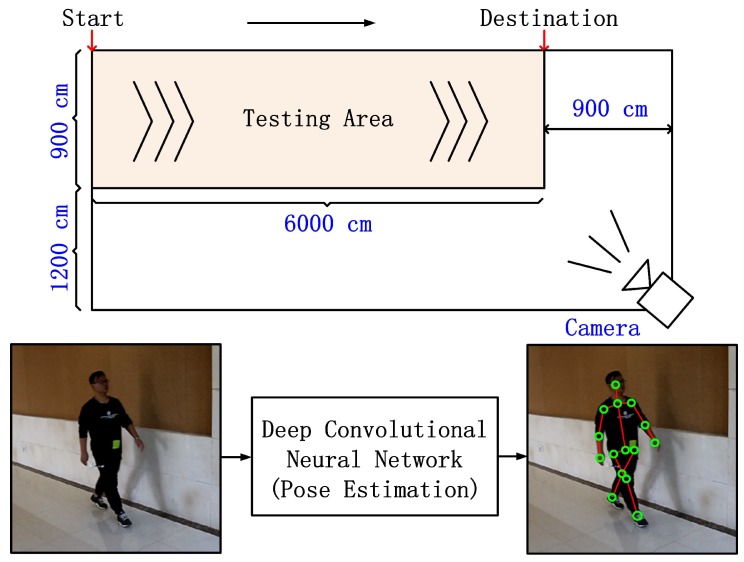
Overview of the capture system and the captured images. The top is the testing field of our database, and the bottom is one frame in a single gait sequence of our database, which is captured from the prescribed walking direction.

**Figure 7:**
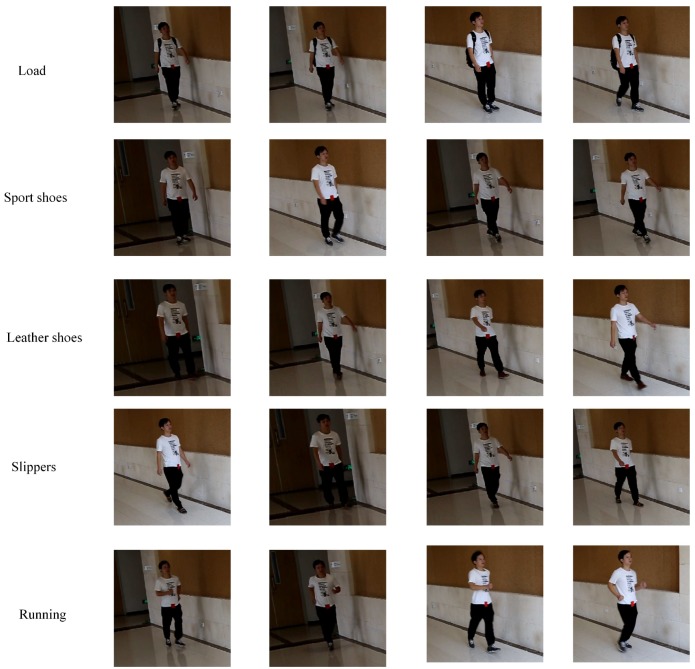
Example frames of Subject 6 in our database.

In our database, we recorded walking and running sequences from 20 individuals (10 males and 10 females). In addition, shoes and carrying condition were used for an exploratory factor analysis of walking recognition. For the first factor, we recorded the three types of shoes for each individual including sport, slipper, and leather shoes. For the second factor, we recorded the sequences where the person carries a backpack of eight kilograms on his/her back. For each impact factor, we recorded 10 cycles of motion for each individual. The camera was placed at 1.20 m above the ground and at 15 degrees relative to the starting point of the walking line. The 3D poses were captured by a unified deep neutral network that presents a two-stage cascaded structure [62]. An overview of our capture system is illustrated in Figure 6. In our database, each individual was asked to walk and run at his or her preferred speed through a straight course. The length of the course was approximately 6 m. We used a 7D camera manufactured by Canon Inc. The original image size and frame rate were 1280×720 pixels and 50 fps, respectively. In order to reconstruct the 3D pose from each frame, each frame was cut to a man-centered size of 256×256. Figure 7 shows example frames in walking sequences of Subject 6. The source codes and data (RGB and 3D skeleton sequences) will be available at our research group website.

Since the proposed features were extracted from the Riemannian manifold, which essentially describes the system of human movement, once the skeleto-muscular structure was influenced by other external factors (such as shoes or baggage), the motor system changed. Therefore, we modeled different factors separately. In the second row of Table 3, we report the recognition accuracies of walking sequences in our database. From the third row to the fifth row are the recognition accuracies with different influence factors. In addition, in the last row of Table 3, the result suggests that each individual also has a unique motion pattern for running. We can see that our features are robust under conditions of many factors.

**Figure 8:**
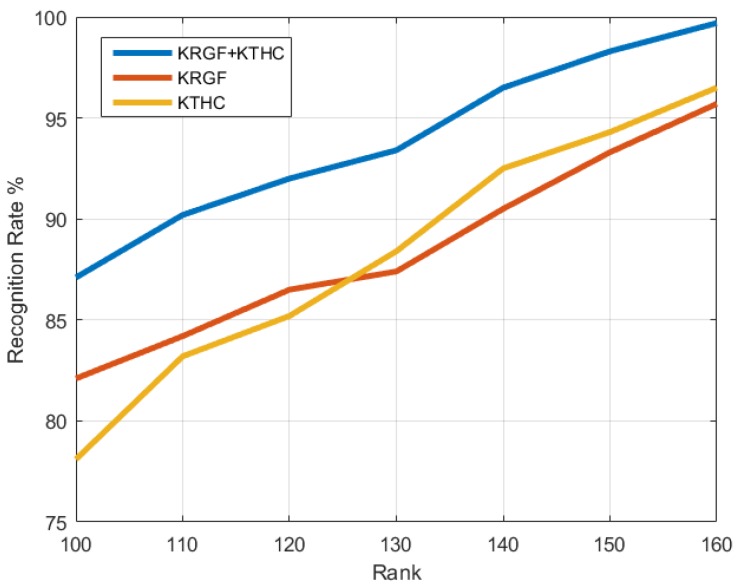
The recognition rate for three proposed features with different feature dimensions: the kernelized Riemannian geometric feature (KRGF), the kernelized Riemannian temporal hierarchy of covariance (KRTHC) feature, and their fusion (KRGF + KRTHC) on the walking sequence in our database.

Figure 8 shows the recognition accuracy of the proposed approach in walking sequences versus feature dimensions. We can see that the fusion feature (KRGF+KRTHC) outperforms both the kernelized Riemannian geometric feature (KRGF) and the kernelized Riemannian temporal hierarchy of covariance (KRTHC) feature by a considerable margin. Specifically, the KRTHC feature can model pose variations within a sequence, but it may ignore the important local differences between individuals. Combining the local features encoded by the KRGF and the large-scale temporal features produced by the KRTHC feature, the proposed approach can simultaneously capture the varying spatial distribution of motion and its temporal variations.

## 5. Conclusions

In this work, we focus on the problem of individual identification from the locomotion sequence. We model the human motion system on the Riemannian manifold, which is an essential characterization of human movement. The aligned Riemannian mean motion sequence provides the common information among different individuals. The spatial state descriptors of individuals can be intuitively expressed by the geometrical distance between a testing sequence and the aligned Riemannian mean motion sequence. In order to take the temporal structure of the human movement into consideration, we modeled the dynamic variations of static geometric features by the temporal hierarchy covariance descriptors. The spatial and temporal features were then efficiently combined by a kernel metric learning method, which was used to obtain the most discriminative information from the feature space. Classification results using the proposed method achieve state-of-the-art results in both walking and running sequences. Combined with pose estimation techniques performed via deep leaning methods, our approach can be used to recognize individuals in real scenarios.

## Figures and Tables

**Table 1:** Recognition rate of all individual recognition methods tested with the CMU Mocap database.

**Methods**	**Ball et al. [53]**	**Ahmed et al. [54]**	**Andersson et al. [55]**	**Jiang et al. [56]**
Accuracy	46.10%	91.20%	92.60%	88.80%
**Methods**	**Preis et al. [57]**	**Sedmidubsky et al. [31]**	**Sinha et al. [58]**	**Ours**
Accuracy	56.00%	81.60%	96.20%	**98.70%**

**Table 2:** Recognition rate of all individual recognition methods tested with the UPCVgait database.

**Methods**	**Ball et al. [53]**	**Preis et al. [57]**	**Kumar and Babu [59]**
Accuracy	14.10%	43.00%	89.20%
**Methods**	**Theodorakopoulos et al. [60]**	**Kastaniotis et al. [61]**	**Ours**
Accuracy	94.80%	96.20%	**97.90%**

**Table 3:** Recognition rate of the proposed method on different sequences in our database.

Feature Dimension	*p* = 100	*p* = 100	*p* = 120	*p* = 130	*p* = 140	*p* = 150	*p* = 160
Walking	87.1%	90.2%	92.0%	93.4%	96.5%	98.3%	99.7%
Load	82.1%	84.3%	88.8%	91.3%	93.1%	96.1%	96.2%
Leather	85.2%	87.5%	90.7%	93.2%	95.0%	97.6%	98.2%
Slippers	84.5%	86.8%	87.3%	91.5%	93.2%	96.9%	97.3%
Running	82.5%	85.6%	88.9%	92.2%	95.5%	96.4%	96.8%
